# Inner Ear Otolith Asymmetry in Late-Larval Cichlid Fish (*Oreochromis mossambicus*, Perciformes) Showing Kinetotic Behaviour Under Diminished Gravity

**DOI:** 10.1038/s41598-017-15927-z

**Published:** 2017-11-15

**Authors:** Ralf Anken, Miriam Knie, Reinhard Hilbig

**Affiliations:** 1 German Aerospace Center, Institute of Aerospace Medicine, Gravitational Biology, Linder Hoehe, D-51147 Cologne, Germany; 2 Institute of Zoology, University of Hohenheim, Garbenstr. 30, D-70599 Stuttgart, Germany; 3 Animal Ecology I, University of Bayreuth, Universitaetsstr. 30, D-95447 Bayreuth, Germany

## Abstract

The inner ears of all vertebrates are designed to perceive auditory and vestibular inputs. Although a tremendous diversity in the inner ear can be found even among bony fishes, the morphologies of the utricle and of the semicircular canals are rather conservative among vertebrates. Fish show kinetoses under reduced gravity (spinning movements and looping responses) and are regarded model organisms concerning the performance of the otolithic organs. Otoliths can be analysed easily because they are compact, in contrast to the otoconial masses of other vertebrates. Here, late-larval *Oreochromis mossambicus* were subjected to 0.0001 × g and 0.04 × g aboard a sounding rocket, their behaviour was observed and morphometrical analyses on otoliths were carried out. Fish swimming kinetotically at 0.0001 × g had a higher asymmetry of utricular otoliths (gravity perception) but not of saccular otoliths (hearing process) than specimens behaving normally at this gravity level (p = 0.0055). Also, asymmetries of lapilli in animals swimming normally at 0.0001 × g were lower than asymmetries in specimens swimming normally at 0.04 × g (p = 0.06). This supports the “otolith asymmetry hypothesis”, an explanation for the susceptibility to kinetosis, particularly concerning the utricular otoliths. It would be interesting to identify processes generating asymmetric otoliths, also in regard to human motion sickness.

## Introduction

In humans (as well as in other vertebrates) diminished gravitational environments such as weightlessness (microgravity, μg) induce changes in the central and peripheral interpretation of sensory input leading to Space Motion Sickness, SMS^1–10^, which is a sensory motor kinetosis normally accompanied by malaise and vomiting. Such a kinetosis can also be observed in a variety of further environments during motion such as involving, e.g., cars, boats, planes, tilting trains, funfair rides, virtual reality and others^8^. It is widely accepted that an explanation for the development of motion sickness is provided by the sensory mismatch theory^11^, also called the intersensory-conflict theory^12^, which means that contradictory visual and vestibular information effects SMS development. In order to provide a reason for mismatching sensory input under diminished gravity, it has been argued that bilateral asymmetry is a common feature at all levels of organisation and that, for postural control, vestibular afferents ought not to be too asymmetric (based on asymmetric inner ear otoliths) in order to stay in the range of the central nervous vestibular compensation of bilaterally different inputs^9,13–16^. According to an earlier hypothesis, developed in 1979^17^ and refined later^18–21^, such possible asymmetries might become disclosed under μg conditions and contribute to the development of SMS.

There is a tremendous diversity among the inner ears of vertebrates^22^ and even among bony fishes^22,23^, but some morphological features are rather conservative. While the pars inferior (e.g., saccule and lagena, auditory end organs) has considerably been changed in developing an auditory function, the pars superior of the labyrinth (utricle and semicircular canals) has remained fairly constant during evolution^24^.

Fishes are established animal models to study vestibular malfunctions. They possess no body-weight related proprioperception to be used for maintenance of equilibrium and thus have to rely on their vestibular system for postural control. Most species like the zebrafish *Danio rerio*, the medaka *Oryzias latipes* and the cichlid *Oreochromis mossambicus* - used in the present study - moreover possess solid ear stones, which can easily be dissected, handled and analysed, whereas other vertebrates including humans have hundreds of thousands of minute stato- (or oto-) conia, which cannot easily be quantified concerning the total mass of an otoconial layer.

Since the occurrence of a kinetosis can be easily observed in the form of so-called looping responses and spinning movements in fish^25–28^, we have shown earlier using fish (late-larval cichlid fish *Oreochromis mossambicus*) as vertebrate model system that such individual animals, which exhibit looping responses and spinning movements at the transfer from 1 × g Earth gravity to diminished gravity during parabolic aircraft flights (0.04 × g, Low Quality Microgravity, LQM) had, statistically, a higher otolith asymmetry as compared to fish swimming normally at experimental conditions^29^. In the course of an experiment under extremely low gravity, i.e., 0.000001 × g (High Quality Microgravity, HQM, ZARM drop-tower at Bremen, Germany), we found out that the ratio of late-larval cichlid fish of a given clutch being susceptible to kinetosis strongly depends on the level of environmental gravity^27^: Kinetosis was observed in more than 90% of the individuals subjected to 0.000001 × g which lead to the conclusion^28^, that this level of environmental gravity is not sufficient to be used by the experimental animals as a cue for spatial orientation. In contrast, a kinetotic behaviour was shown by less than 25% of the animals subjected to 0.04–0.05 × g in the course of parabolic aircraft flights^29^. Such experiments, always using late-larval *O. mossambicus*, led us to the assumption that the level of otolith asymmetry resulting in kinetotic behaviour may vary depending on the environmental gravity vector, and we were prompted to test this hypothesis by subjecting this established fish model to two levels of diminished gravity aboard a (centrifuge-equipped) sounding rocket.

Indeed, the experiment carried out here showed, that animals still behaving normally at strongly reduced gravity possessed a particularly low level of otolith asymmetry. Vice versa, animals exhibiting a somewhat higher otolith asymmetry were still able to swim normally at a level of gravity not that much reduced. Otolith asymmetry was especially high in animals swimming kinetotically as compared with those of normally swimming specimens under strongly diminished gravity.

Overall, our results clearly support the hypothesis that otolith asymmetry is a major source of kinetosis susceptibility in fish.

## Results

**Figure 1:**
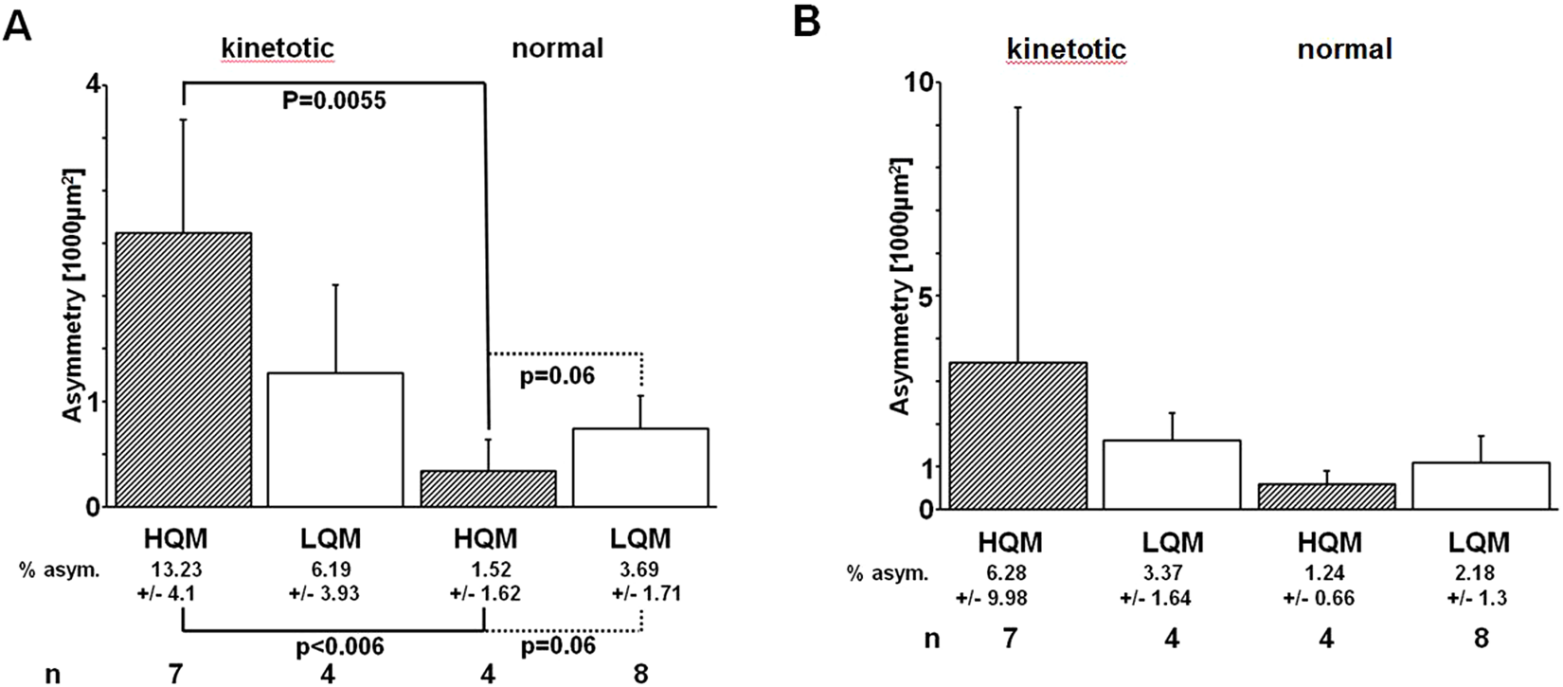
Otolith asymmetry of experimental animals. Mean absolute (bars) and percentual (“% asym.”, see the text below the X-axes) otolith asymmetry of late-larval cichlid fish, who either swam kinetotically or normally under two levels of diminished gravity (HQM, High Quality Microgravity: 0.0001 × g; LQM, Low Quality Microgravity: 0.04 × g) during the 6 minutes lasting microgravity phase of a sounding rocket flight. Where no p-values are indicated, the level of significance was p > 0.1. Error bars and +/− signs indicate standard deviations. (**A**) Lapilli. (**B**) Sagittae.

Under HQM (0.0001 × g), approximately 30% of all animals swam kinetotically, approximately 17% swam normally. Under LQM (0.04 × g), kinetotic and normal swimmers amounted to approximately 8% and 17%, respectively (see Fig. 1 for absolute numbers).

The remaining specimens did not show either a clear kinetotic or normal, gravity-based behaviour, but rested, performed a dorsal/ventral light response or disoriented movements. Since these types of behaviour cannot comprehensively be related to vestibular issues addressed in this study, such animals were not subjected to further analysis (please consult the section on methods for details).

Following the individual analysis of the otoliths, a normal-distributed overall variance of their surface areas was observed as was the case in earlier studies^28,29^. However, utricular otolith asymmetry (both in absolute values as well as in percentage) was especially high in animals which swam kinetotically under HQM in comparison to normal swimming specimens (Fig. 1, p = 0.0055). There was a trend that animals swimming normally under HQM had lower asymmetries than specimens swimming normally under LQM (p = 0.06). Specimens swimming kinetotically under LQM did not show a significant difference in utricular asymmetry as compared to normally behaving specimens. Concerning sagittal asymmetry, no statistically relevant results were found (Fig. 1).

## Discussion

Asymmetry of fish otoliths has been extensively investigated in fisheries sciences as a useful bioindicator of exogenous environmental stressors^30^. By far fewer investigations have been focused on otolith asymmetry in a functional context. In a series of highly interesting investigations, fish otolith mass asymmetry (mostly using sagittae) was studied regarding their role in acoustic functionality^31–33^. An important finding was that symmetrical fish do not have a “principal” ear, a clear handedness, in which the heavier otoliths are consistently located on the one or the other side^31^. The otolith growth rate may even vary during the lifespan of an individual animal, otolith asymmetry thus is not constant, but does not exceed some 20% between a left and a right otolith asymmetry^31^. In flatfishes, however, otolith asymmetries seem to reveal a species-specific handedness, but, theoretically, a one-sided saccular prevalence has no heavy implications concerning the processing of sound^33^. It matters, obviously, the lifestyle of a fish, if a particular otolith asymmetry is detrimental or not.

Regarding otolith mass asymmetry in the context of acoustic functionality, the question was forwarded, if a specific mechanism exists which maintains otolith asymmetry below a critical value^31^; it remains to be answered. Since stimulus dependence is a general feature of developing sensory systems, it has frequently been shown using fish that especially utricular otoliths can be well assessed as test masses with regard to their function as gravity sensors. When the environmental gravity vector is altered, the growth of otoliths is adjusted in order to adapt to the new gravitational environment^13,34–38^.

We have shown earlier that a unilateral vestibular nerve dissection stops calcium incorporation into the otoliths of the lesioned side in neonate swordtails *Xiphophorus helleri* ^15^ and that calcium incorporation resumes after the vestibular nerve has regenerated (unpublished own results; no data on developing cichlids available). Furthermore, we have demonstrated that a development of larval cichlids under 3 × g hypergravity slows down otolith growth and additionally renders animals with a much lower otolith asymmetry as compared to 1 × g controls^13^. We do not know how a mechanism adjusting otolith masses may work, but it is possible that this or a similar mechanism maintains a level of asymmetry as low as necessary regarding auditory or gravity sensing functionality. This feedback hypothesis is, however, not generally accepted^31^, and we ourselves came to a conclusion that things are not that easy, since the regulation of otolithic calcium incorporation is guided only in part via the vestibular nerve, but, in part, via a further pathway, which remains hitherto elusive^39^.

The present study gives evidence that the appearance of kinetotic behaviour in late-staged larval cichlid fish is correlated with the degree of utricular otolith asymmetry. Certainly, an evaluation of the surface of otoliths as carried out here is a very indirect measure to obtain a cue on the underlying otolith mass, since particularly lapilli show a strong three-dimensional shape in many species of fish. This is also true for asterisci of the lagenae, why in fisheries sciences sagittae are preferentially used for quantitative, comparative investigations, not only because sagittae are comparatively large and hence can be easily dissected. However, as has been stated in the methods section, utricular otoliths of late-larval cichlids are almost planar and the calcium content of such otoliths as well as their volumes are highly significantly correlated with their surface area. This surface area has been measured in the present study and is used as a proxy for otolith mass, albeit to be interpreted with some caution.

In principle, a possible role of the semicircular canals in generating kinetoses cannot be excluded and needs to be considered here, because the semicircular organs play a decisive role in postural and visual stabilisation. It has been shown in some aquatic vertebrates such as in tadpoles of the clawed toad *Xenopus laevis* that canal sensitivity increases with both a larger canal lumen and circuit radius, and that a minimum dimension for semicircular canals is necessary in order to gain full canal operation^40^. An angular vestibulo-ocular reflex (aVOR), which is a response of semicircular canal stimulation, develops rather late in zebrafish, medaka and goldfish^41^, but such vestibulum-induced eye movements can be evoked in zebrafish larvae by both static tilts and dynamic rotations^42^, which allow a stabilisation of retinal images during movements and thus perform the task of the later developed aVOR. There are no data available on the onset of operation of the aVOR in cichlids, but the roll-induced static vestibulo-ocular reflex is fully operational already in stage 14–16 cichlids^43^ and thus in late-larval animals as used in the present study.

Hence, a contribution of the semicircular canals or/and circuits other than those related to the macular organs to kinetosis cannot be fully excluded.

Certainly, the developmental stage of the neuronal circuitry underlying kinetosis susceptibility also plays a prominent role. It has been shown using experimentally generated three-eared frogs^44^ that mechanisms may act on the refinements of vestibular projections, resulting in a mismatched perception of gravity^44^. In case that the transplanted ear was in alignment with the native ear, the tadpoles swam normally; in case a transplanted ear was rotated by 90 degrees, the swimming was aberrant and similar to the swimming mode of such animals, where one native ear had been removed^44^ (which certainly represents an asymmetry). In consequence, bilateral symmetry in sensory epithelia is required for proper postural control and normal swimming^44^.

Several observations in fact speak in favour of the assumption that rather the otolithic organs are responsible for generating motion sickness^2,3,18,45^. However, in zebrafish, an abnormal swimming behaviour was induced by a vestibular dysfunction of the semicircular canal ducts, observed in experiments testing bmp2b in mutant swirl embryos^46^. When making sudden changes in direction, e.g., when an animal is disturbed, its dorsoventral orientation is lost resulting in a kind of somersaulting, clearly indicating a defect in the detection of angular motion^46^. The sudden spinning movements and looping responses observed in late-larval cichlids at the onset of diminished gravity, however, are a completely different kind of behaviour and angular detection should not be disturbed in resting fish at a sudden decrease of environmental gravity.

The percentage of kinetotically swimming individuals was considerably higher under HQM (0.0001 × g) than under LQM (0.04 × g). This finding is in full agreement with earlier results using likewise late-staged larval cichlids, according to which the level of the residual gravity during experiments at reduced gravity is directly correlated with the occurrence of kinetoses.

Under parabolic flight microgravity, the residual gravity level of approximately 0.04–0.05 × g only effects a kinetosis in rather few animals of the same clutch^29^. In contrast, almost all (>90%) individuals having been kept at 0.000001 × g (drop-tower at ZARM, Bremen, Germany) showed a kinetotic behaviour^28^. Obviously, interpreting the data of our present study, a residual gravity of only approximately 0.0001 × g was sufficient for 17% of the fish of the given clutch to be used for postural control. Under LQM, also around 17% of all animals showed a normal, gravity-related behaviour. This result also is in line with earlier experiments carried out under LQM during parabolic flights^29^.

Otolith analyses on saccular stones (sagittae) did not yield any significant findings comparing the data from the various groups investigated. Since sagittae play their role rather in hearing than in detecting gravity^47^, the result met our expectations.

Asymmetry of utricular otoliths (lapilli, gravity detection^47^), however, was significantly higher in kinetotically vs. normally swimming animals kept under HQM. Respective differences in lapilli asymmetry of animals maintained under LQM were not found. The latter result indicates that an otolith asymmetry does not necessarily play a crucial role in postural control, as long as the remaining gravity level is sufficient to be perceived by the animal with its given otolith asymmetry, which is in full accordance with earlier results obtained in the course of parabolic flight studies^29^. Under HQM, however, a high otolith asymmetry obviously is detrimental, causing an animal to swim kinetotically. At such a low level of gravity, an otolith asymmetry beyond a threshold yet to be found, seems not anymore to be feasibly compensated for by the nervous system (integrating further cues such as visual ones). We also found (p = 0.06) that otolith asymmetry is lower in animals swimming kinetotically under HQM as compared with normal swimmers under LQM. This result indicates that normal swimming under HQM requires a particularly low otolith asymmetry.

In conclusion, our findings support the assumption, that utricular otolith asymmetries increasingly come into detrimental operation, when the level of gravity is lowered. It would be interesting to experimentally decrease or increase otolith asymmetry (by which means ever; an extreme asymmetry could be achieved by hemilabyrinthectomy), let animals compensate at 1 g Earth gravity and then carry out another experiment under diminished gravity. We then would expect that animals with a very high utricular asymmetry would go kinetotic already at slightly diminished gravity. Also, it would be of interest to investigate if there is a kind of threshold of kinetosis susceptibility, correlated with a particular otolith asymmetry.

Although it is not possible to directly transfer our considerations to terrestrial vertebrates since maintenance of postural control does not involve the very same organs and mechanisms among the various taxa, our results clearly indicate that utricular otolith asymmetry plays a prominent role in susceptibility to kinetosis and a disorder in spatial orientation. Overall, our results support the “otolith asymmetry hypothesis” as an explanation for kinetosis susceptibility.

## Methods

TEXUS sounding rocket flights are a means to accomplish experiments at microgravity lasting around 6 minutes. A definition of the term “microgravity” and a description of techniques to acquire this condition have been published earlier^48^. On behalf of the European Space Agency ESA, these rockets are launched at ESA’s site at Kiruna, Sweden (Esrange).

### Animals

Late-stage larval cichlid fish siblings (*Oreochromis mossambicus*, Perciformes) were used. They had reached developmental stage 23 (24 days post fertilisation at 22 °C^49^), when all fins except the pelvic fins have rays, the vestibular system is operational and the swimming performance is fully developed. Thus, they behave like adult specimens, but are small enough to be kept in small aquaria as required to be implemented into a sounding rocket, where only very limited space is available.

### Flight experiment

The small late-staged cichlid fish were individually housed within cylindrical tanks (diameter: 30 mm; height: 50 mm; volume ca. 35 cm^3^) milled out of Makrolon® blocks. These tanks were assembled to modules of 8 and each single tank was equipped with a pressure compensation unit. At the bottom of the modules an illumination foil was attached (electro-luminescence foil ELF, Lumitec AG, Gais, Switzerland). Via an observation window in each module the behaviour of the fish could be recorded permanently by CCD-cameras (Sony XC ST70), written on a flash card recorder. In the course of the TEXUS rocket flight, 24 animals were kept in tanks fitted to a fixed platform receiving 0.0001 × g (High Quality Microgravity, HQM^50^), whereas another 48 specimens were subjected to 0.04 × g (Low Quality Microgravity, LQM^50^) in tanks mounted on a rotating platform (centrifuge). Further details on the hardware have been published earlier^51^.

### Post-flight procedures and data collection

After flight, the animals were, in order to ameliorate suffering, deeply anesthetised using tricain-s (Western Chemical Inc., Ferndale, USA), killed by pithing and transferred to 90% ethanol for storage.

Specimens, which unambiguously showed the same swimming behaviour throughout the entire phase of diminished gravity during the flight were qualitatively classified as either swimming kinetotically or normally^27–29^. Kinetotic individuals showed looping responses (i.e., swimming around in rapid circles) or spinning movements (turns along the body axis) while normally swimming ones showed a straight forward swimming behaviour or swam along the walls, with occasional turns. All other animals, exhibiting types of behaviour which could not be classified unambiguously as either “normal” or “kinetotic”, were not subjected to detailed further analyses of the otoliths. These other individuals did not move at all, oriented themselves to the light source or showed escape responses etc., which cannot be directly related to vestibular issues which were in the focus of our interest.

As has been observed earlier^29^, only some animals of a given clutch or experimental group swim either clearly kinetotically or clearly normally at decreased gravity. This depends on the level of gravity administered and on the developmental stage of the animals used^52^ as well as on a variety of further factors such as the visual performance of an individual eventually resulting in a dorsal/ventral light response behaviour, on the level of arousal of an individual sample and others. Consequently, we employed here as many specimens as technically feasible in order to collect a number of unambiguously kinetotically and normally swimming fish sufficient to carry out reliable statistical analyses.

Subsequently, left and right utricular and saccular otoliths (i.e., lapilli – gravity perception^47^ – and sagittae – hearing process^47^ – respectively) were dissected from the stored specimens and mounted pairwise (with that side up, which is situated proximal to the sensory epithelium; this side is characterised by shallow grooves) in glycerine on microscopical slides. Using a computer-based image analysing system (Axioscope Imager 1, Axiovision 4.6 software, both Zeiss, Germany), the size of otoliths (i.e., the two-dimensional projection of the surface area) was determined planimetrically and the absolute as well as the percentual bilateral asymmetry (differences in size between the right and the left stones) was calculated.

Both percentual asymmetry as well as absolute asymmetry were evaluated in the present study, since the former takes into account the size of otoliths, i.e., a high value of absolute asymmetry needs not to be crucial for the organism if the individual otoliths were large, whereas a high value of percentual asymmetry should be crucial – independent of the absolute sizes of the otoliths.

Otoliths of larval cichlid fish are too small to be weighed. Earlier, methodological studies have shown that the calcium content of the otoliths of larval cichlid fish (measured by inductively coupled plasma mass spectrometry)^37^ as well as the otoliths’ volume (determined by a three-dimensional reconstruction using laser scanning microscopy)^53^ is significantly (p < 0.0001) correlated with the otoliths’ surface areas (likely, because otoliths from larval cichlids are almost planar). Since it is methodologically much easier and faster to determine the surface area of larval fish otoliths than employing inductively coupled plasma mass spectrometry and/or laser scanning microscopy, we routinely use the former technique.

### Statistical analysis

All analyses, dissection of otoliths and their measurements were scored blind to avoid observer bias. Cumulative statistics of all otoliths were performed calculating mean, standard deviation, median, minimum and maximum. The respective values were within the range of 2.0 σ. Due to their respective behaviour, in total 11 exclusively normally swimming and 12 exclusively kinetotically behaving fish were selected for further calculations. Statistics of these groups were based on Student’s t test with a 95% confidence interval. See caption of figure for sample sizes.

### Ethics

All issues concerning maintenance, handling, transport, experimental procedure in the course of the TEXUS-campaign, sacrifice and analysis of fish were carried out in full accordance with the German and Swedish Laws on Animal Welfare (names of relevant IACUCs: Regierungspräsidium Tübingen and Ansökan om tillstand att använda försöksdjur: Diariennummer/AZ 31-9495/07 and A3-08). The named IACUCs have specifically approved the present study.

### Data availability

The datasets generated during and/or analysed during the current study are available from RH on reasonable request.
